# Chronic effects of blast injury on the microvasculature in a transgenic mouse model of Alzheimer’s disease related Aβ amyloidosis

**DOI:** 10.1186/s12987-021-00301-z

**Published:** 2022-01-10

**Authors:** Alexander T. Clark, Eric E. Abrahamson, Matthew M. Harper, Milos D. Ikonomovic

**Affiliations:** 1 Geriatric Research Education and Clinical Center, VA Pittsburgh Healthcare System, University Drive C, Pittsburgh, PA 15240 USA; 2 Department of Neurology, University of Pittsburgh School of Medicine, 3471 Fifth Ave, Pittsburgh, PA 15213 USA; 3 Department of Psychiatry, University of Pittsburgh School of Medicine, Thomas Detre Hall of the WPH, Room 1421, 3811 O’Hara Street, Pittsburgh, PA 15213-2593 USA; 4 The Iowa City VA Center for the Prevention and Treatment of Visual Loss, 601 Hwy 6 West, Iowa City, IA 52246 USA; 5 Department of Ophthalmology and Visual Sciences and Biology, University of Iowa, 200 Hawkins Dr, Iowa City, IA 52242 USA

**Keywords:** Alzheimer’s disease, Amyloid, Astrocyte, Blast injury, Blood–brain barrier, NVU, Neurovascular unit, Pericyte, Traumatic brain injury

## Abstract

**Background:**

Altered cerebrovascular function and accumulation of amyloid-β (Aβ) after traumatic brain injury (TBI) can contribute to chronic neuropathology and increase the risk for Alzheimer’s disease (AD). TBI due to a blast-induced shock wave (bTBI) adversely affects the neurovascular unit (NVU) during the acute period after injury. However, the chronic effects of bTBI and Aβ on cellular components of the NVU and capillary network are not well understood.

**Methods:**

We exposed young adult (age range: 76–106 days) female transgenic (Tg) APP/PS1 mice, a model of AD-like Aβ amyloidosis, and wild type (Wt) mice to a single bTBI (~ 138 kPa or ~ 20 psi) or to a Sham procedure. At 3-months or 12-months survival after exposure, we quantified neocortical Aβ load in Tg mice, and percent contact area between aquaporin-4 (AQP4)-immunoreactive astrocytic end-feet and brain capillaries, numbers of PDGFRβ-immunoreactive pericytes, and capillary densities in both genotypes.

**Results:**

The astroglia AQP4-capillary contact area in the Tg-bTBI group was significantly lower than in the Tg-Sham group at 3-months survival. No significant changes in the AQP4-capillary contact area were observed in the Tg-bTBI group at 12-months survival or in the Wt groups. Capillary density in the Tg-bTBI group at 12-months survival was significantly higher compared to the Tg-Sham control and to the Tg-bTBI 3-months survival group. The Wt-bTBI group had significantly lower capillary density and pericyte numbers at 12-months survival compared to 3-months survival. When pericytes were quantified relative to capillary density, no significant differences were detected among the experimental groups, for both genotypes.

**Conclusion:**

In conditions of high brain concentrations of human Aβ, bTBI exposure results in reduced AQP4 expression at the astroglia-microvascular interface, and in chronic capillary proliferation like what has been reported in AD. Long term microvascular changes after bTBI may contribute to the risk for developing chronic neurodegenerative disease later in life.

## Background

Traumatic brain injury (TBI) is considered a risk factor for chronic neurodegenerative disorders including Alzheimer’s disease (AD), Parkinson disease (PD), and chronic traumatic encephalopathy (CTE) [37, 75, 77, 86]. The pathobiological changes underlying this phenomenon are not well-understood. One hypothesis is that TBI induces or accelerates accumulation of aggregation-prone molecules such as amyloid-β (Aβ) peptides, hyper-phosphorylated tau protein, alpha-synuclein, and transactive response DNA-binding protein 43 (TDP-43) [55, 60, 130]. Pertaining to the risk for AD following TBI, an injury-induced imbalance between Aβ production and clearance can promote accumulation of Aβ in the brain [20, 58, 109] which can contribute to, and be enhanced by, vascular changes involving impaired blood–brain barrier (BBB) function and dysregulation of cerebral blood flow [3, 53, 54, 57, 67, 118, 136]. Because Aβ- and vascular changes could contribute to poor recovery after a TBI and, according to the two-hit vascular hypothesis, promote chronic neurodegeneration in AD [92, 136], they are attractive targets for development of therapy interventions and biomarkers in both conditions [23, 34, 54, 66, 104, 120, 122].

Cerebrovascular homeostasis is regulated by the combined actions of endothelial cells, mural cells (capillary pericytes and smooth muscle cells), glial cells, and neuronal activity [54, 74, 123]. Pericytes have multiple roles in microvascular function [132] including modulation of capillary diameter and cerebral microcirculation [41, 45, 68, 69, 91, 99] and maintenance of endothelial junctional proteins that form the BBB [16, 123]. The barrier function of endothelial cells and pericytes is influenced by astrocytes which secrete (and uptake) cytokines, growth factors, and neurotransmitters [1, 54, 123], and interact with pericytes in regulating neurovascular coupling [54, 88]. In the aftermath of a brain injury, both pericytes and astrocytes are involved in reparatory processes [9, 21, 23] and are capable of clearing Aβ by an apolipoprotein E/low-density lipoprotein receptor-related protein 1 (LRP-1) mediated mechanism [6, 76, 82, 124, 126] and secretion of Aβ-degrading enzymes [108]. Thus, brain injury-induced dysfunction or loss of either cell type could impair these mechanisms and contribute to brain Aβ accumulation in conjunction with microvascular dysregulation.

Clinical studies, and experimental studies in animals and in vitro models, reported that exposure to explosive blasts can result in acute dysfunction or loss of pericytes and astrocytes as well as altered BBB permeability [2, 5, 7, 13, 30, 35, 36, 38, 39, 47–52, 61, 63–65, 71–73, 78, 79, 81, 83, 84, 105, 112, 116, 129, 134]. However, the effects of blast TBI on brain Aβ concentration and deposition have been assessed in only a few studies and at acute time points after injury exposure. In wild type rodents, endogenous murine Aβ was lower acutely in animals exposed to a single low-level blast TBI (bTBI) compared to sham animals [27], while transgenic AD mice exposed to repetitive low-level bTBI with 4 weeks survival had lower levels of soluble and insoluble Aβ but no change in Aβ plaque load compared to sham mice [100]. These findings were unexpected due to previous reports of increased Aβ concentration and deposition in brains of young adults within hours to days after severe TBI [29, 58], and higher Aβ concentrations at 3 weeks after controlled cortical impact injury in human Aβ knock-in mice compared to sham mice [3], suggesting that blast TBI and blunt force TBI may have differential effects on Aβ production, accumulation and/or clearance. To gain insight into the unexplored chronic effects of blast TBI on Aβ accumulation and microvascular changes, the current study exposed transgenic (Tg) APP/PS1 mice to bTBI at the age prior to their onset of Aβ deposits, and at 3 months and 12 months after injury evaluated Aβ deposition, pericyte numbers, astrocytic end-foot/capillary interactions, and capillary densities. We hypothesized that bTBI would produce chronic alterations in the capillary network, pericytes, and perivascular astrocytes in both genotypes, with Tg mice showing exacerbated changes and enhanced burden of Aβ pathology.

## Methods

### Experimental animal groups, genetics, and interventions

The Iowa City Department of Veterans Affairs and the University of Pittsburgh Institutional Animal Care and Use Committees approved all investigative procedures. The study used adult female transgenic APPswe, PS1ΔE9 (B6C3Tg(APPswe,PSEN1dE9)85Dbo/Mmjax, The Jackson Laboratory, MMRRC Stock No: 34829-JAX | APP/PS1) double mutant mice (referred to as APP/PS1 or Tg mice in this report) and their littermate B6C3F1/J (Stock No: 100010, Jackson) wild-type controls (Wt). The APP/PS1 mice harbor a ‘humanized’ APP gene driven by the prion promoter, with two mutations which are linked to familial AD [25, 90]. The Swedish mutation enhances β-secretase activity while the human presenilin gene with the 9th exon removed enhances gamma secretase processing of APP. These two mutations and constitutive APP overexpression result in enhanced amyloidogenic APP processing, overproduction of Aβ, and progressive Aβ deposition in brain parenchyma and vasculature. The APP/PS1 mice exhibit Aβ pathology at 4 to 6 months of age, and progressively develop more severe parenchymal and vascular amyloid deposits at older ages [59]. This pathology appears to be driven by overall increases in Aβ peptides as well as a shift in the Aβ40:Aβ42 ratio due to larger increases in Aβ42 compared to Aβ40 forms [59]. The Wt mice used in our study are the adult female founder mice of the APP/PS1 transgenics and harbor murine APP and PS1 genes that are driven by their endogenous promoters; these Wt mice do not have human APP and do not exhibit Aβ accumulation and therefore serve as the amyloid-negative control.

Each genotype was randomly stratified into two exposure groups: anesthesia plus blast exposure (referred to as bTBI) or anesthesia without bTBI exposure (referred to as Sham). Sham or bTBI exposure occurred at approximately 3 months of age (range: 76–106 days) for all mice included in the experiment. The mice were then sacrificed after either 3-months survival (around 6 months of age; range: 165–189 days) or 12-months survival (around 15 months of age; range: 442–455 days). We subsequently define age for each experimental group as the post-exposure “survival age” for the remainder of the present study. Accordingly, there were 24 mice in the study, with 3 mice analyzed in each of the 8 experimental groups (Tg bTBI and Tg Sham at 3-months and 12-months survival, and Wt bTBI and Wt Sham at 3-months and 12-months survival).

### Blast injury induction

The blast pressure wave was generated in an enclosed blast chamber which was divided in two parts, with a 13-cm diameter opening between the chamber halves, as described previously [43, 44, 89]. A Mylar membrane (Mylar A, 0.00142 gauge; Country Plastics, Ames, IA) was placed over the opening between the two parts of the blast chamber. One side of the tank remained unpressurized and contained a padded polyvinyl chloride (PVC) protective restraint for positioning of an anesthetized mouse 30 cm from the Mylar membrane [43, 44, 89]. To create the blast wave, compressed room air was pumped into the pressurized side of the tank to 20 psi, the pressure at which the membrane ruptures. Using this model, a complex blast wave without a negative pressure component was produced as described [43] with the following characteristics: 137.8 ± 1.3 kPa (~ 20 psi) peak overpressure at the point of exposure (i.e., the head, the body of the animal was shielded from the blast wind) and a 10- to 15-ms blast duration [43, 89]. The pressure was calculated by using a sensor 1 cm in diameter placed directly below the head of the mouse [89]. Prior to blast wave induction, mice were anesthetized by an intraperitoneal (IP) injection of a combination of ketamine (0.03 mg/g body weight) and xylazine (0.005 mg/g body weight). Mice were positioned within the unpressurized half of the blast chamber with the left side of the head oriented toward the source of the blast wave. Only the head of the mouse was exposed to the blast wave, with the rest of the body shielded from the blast wind. The head of the mouse was unrestrained during the blast wave exposure, allowing for free rotation without contact with the cradle. Sham mice underwent the same anesthesia procedure and were placed in the blast chamber but did not receive a blast exposure. After bTBI exposure or Sham procedure, mice were placed on a heating pad to facilitate recovery from general anesthesia and to maintain a body temperature of 37 ± 0.5 °C. Xylazine anesthesia was reversed with yohimbine chloride (0.001 mg/g, IP) to facilitate the recovery from anesthesia. Mice in the Sham and bTBI groups received analgesic via subcutaneous injection (0.1 mL/20 g body weight) of buprenorphine (0.003 mg/mL) immediately after recovery from either procedure.

### Tissue processing

At the end of the experiment, mice were anesthetized and killed by transcardial perfusion with 0.9% saline which also washed blood from the brain vasculature. After perfusion, the skull was opened to extract the brain, and the left hemisphere was placed into 4% paraformaldehyde fixative for 48 h at 4 °C after which it was infiltrated with graded sucrose solutions to allow for frozen sectioning. The brain hemisphere was then cut in the coronal plane using a freezing sledge microtome to create 40 µm-thick tissue sections. Sections were stored in a cryopreservation solution [131] at − 20 °C until use. Two coronal sections at the level of the dorsal hippocampus were selected randomly and included in the analysis of each mouse.

### Immunohistochemistry and histology

Tissue sections were placed free-floating in 0.1 M potassium phosphate-buffered solution (KPBS), pH 7.4, for 24 h to wash off the cryoprotection solution. Sections were then washed in Tris-buffered saline solution (TBS) before incubation in 0.5% sodium borohydride made in TBS for 20 min. After again washing in TBS, the sections were incubated in 3% normal goat serum (NGS, Sigma, catalogue #G9023) in TBS at room temperature for 30 min, followed by two rinses in 1% NGS in TBS at room temperature for 10 min each. The sections were then incubated with primary antibodies specific to the experimental arm. Sections being analyzed for pericytes were incubated at 4 °C for 24 h in mouse monoclonal IgG raised against PDGFRβ (clone D-6, Santa Cruz, catalogue #sc-374573, lot #B2118) diluted 1:250 in 1% NGS made in TBS with Triton, and Lycopersicon Esculentum (Tomato) Lectin (LEL, Vector Laboratories, catalogue #DL-1177, lot #ZE0131) conjugated to a 594 nm-emitting fluorophore and diluted 1:200 in 1% NGS TBS with Triton [10, 103, 110]. Sections included in the astrocytic end-feet analysis were processed under the same conditions as the pericyte marker, but used a mouse monoclonal IgG generated against AQP4 diluted 1:1000 in 1% NGS made in TBS with Triton (clone 4/18, Santa Cruz, catalogue #sc-32739, lot #D2318), and Solanum Tuberosum (Potato) Lectin (STL, Vector, catalogue # B-1165-2, lot #ZC0519) conjugated to biotin and diluted 1:200 in 1% NGS made in TBS with Triton [46, 93]. After primary antibody/lectin cocktail incubation, sections were rinsed in TBS and incubated in goat anti-mouse IgG secondary antibodies (to mark primary antibodies) and streptavidin conjugated to a 488 nm-emitting Alexa fluorophore (streptavidin-Alexa488, to mark STL; Life Technologies, Eugene, OR, catalogue #S323356, lot #1902487) diluted 1:250 in 1% NGS made in TBS with Triton for 90 min at room temperature. The secondary antibodies included goat anti-mouse IgG conjugated to Alexa488 (pericyte studies, Jackson ImmunoResearch, catalogue #115-545-146, lot #138610) and goat anti-mouse IgG conjugated to a 594 nm-emitting Alexa fluorophore (astrocytic end-feet study, Jackson, catalogue #115-585-146, lot #136295) in 1% NGS made in TBS with Triton. Finally, the sections were rinsed in TBS, mounted on glass slides, and coverslipped using 4′,6-diamidino-2-phenylindole (DAPI) based medium (Vector, catalogue #H-1500) and stored in darkness at 4 °C until imaging [62]. The choice of fluorophore combinations for the multi-fluorescence studies was determined empirically. For immunohistochemical staining of Aβ, sections were rinsed in TBS and then incubated in 80% formic acid for two minutes. Endogenous peroxidase activity was inhibited by incubating the tissue in 1.5% H_2_O_2_ made in TBS for 30 min. Sections were then rinsed in TBS and incubated in 5% NGS made in TBS, rinsed, and incubated overnight in mouse monoclonal IgG raised against Aβ diluted 1:3000 in 1% goat serum made in TBS (clone 6E10, BioLegend catalogue #803002, lot #B198896) at 4 °C. Sections were then washed in TBS and incubated in biotinylated goat anti-mouse IgG (Jackson, catalogue #115-065-146, lot #128250) in 1% goat serum made in TBS for 1 h, washed in TBS, and reacted using the avidin–biotin method (ABC Elite kit, Vector Laboratories). The color reaction was developed using a 0.05% solution of nickel-enhanced 3,3-diaminobenzidine tetra-hydrochloride and 0.03% H_2_O_2_. Sections were then dehydrated in alcohols, cleared in xylenes, and coverslipped with Permount (Fisher). In all experiments using mouse monoclonal antibodies, control tissue sections were processed as described above but without the primary antibody (primary delete). No non-specific staining was observed in these control sections (not shown).

Cyano-PiB (2-(4′-methylaminophenyl)-6-cyanobenzothiazole) histofluorescence was performed as described previously [56]. Tissue sections were incubated in 10 μM cyano-PiB for 45 min in dark conditions at room temperature. Sections were then dipped three times in potassium phosphate buffer, incubated for 1 min in fresh potassium phosphate buffer, and coverslipped with Fluoromount-G (SouthernBiotech, Birmingham, AL, catalogue #0100-01).

### Random sampling scheme of the cerebral cortex

Image stacks were acquired using an Olympus BX-51WI upright microscope equipped with an Olympus DSU spinning disk confocal, a super-corrected 65× Olympus Plan Apo N 1.42 numerical aperture oil immersion objective, a MBF CX9000 front mounted digital camera (MBF Bioscience), a BioPrecision2 XYZ motorized stage with linear XYZ encoders (Ludl), and filters for laser wavelength output of 405 nm (to visualize DAPI), 488 nm (to visualize the Alexa 488 fluorophore), and 594 nm (to visualize the Alexa 594 fluorophore). All z-stacks were obtained using an optical section separation interval (z-interval) of 0.25 µm. Two 25 µm-thick (post-processing shrinkage of original 40 µm-thick sections) sections were chosen per mouse using the previously described criteria. Using unbiased stereological principles on StereoInvestigator (MBF), five optical disectors per hemi-section were examined within the cortex using the central sulcus and rhinal fissure as dorsal and ventral borders, respectively.

### Amyloid burden (% area)

**Fig. 1:**
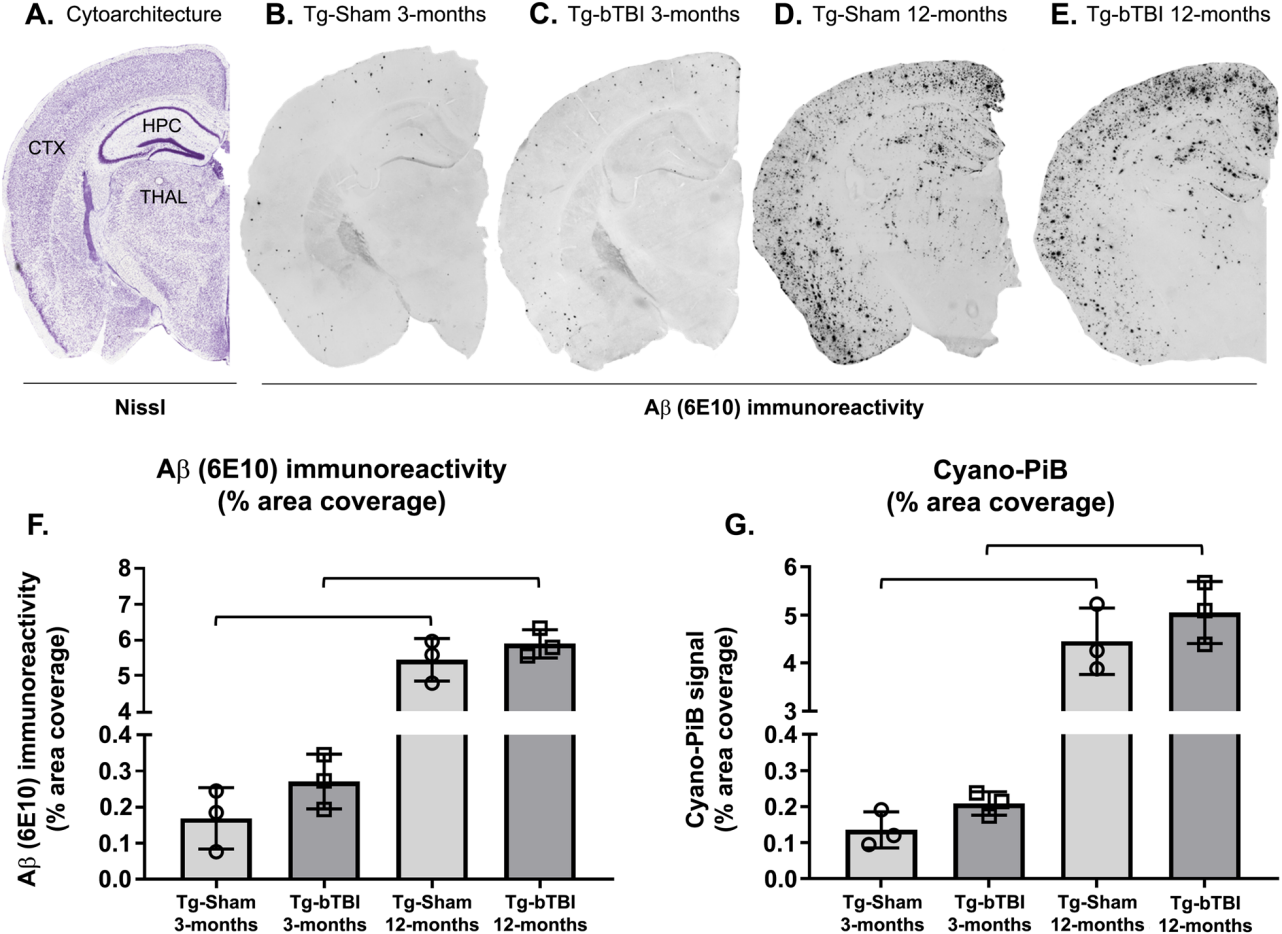
Cortical Aβ load after bTBI in APP/PS1 mice at 3-months and 12-months survival. **A**–**E** A whole hemisphere coronal section stained for Nissl substance (obtained from the Allen Brain Atlas) illustrates the cytoarchitecture of the cortical area targeted (**A**; purple cresyl violet signal) and Aβ-immunostained sections illustrating Aβ pathology load (**B**–**E**) in transgenic mice undergoing Sham or bTBI exposure and evaluated at 3-months survival (**B** and **C**, respectively) or at 12-months survival (**D** and **E**, respectively). **F**, **G** Graphs showing percent area coverage of Aβ-immunoreactive deposits (**F**) and cyano-PiB labeled amyloid pathology (**G**). Wild type mice are not shown as they were free of Aβ immunolabeling and cyano-PiB fluorescence. CTX, neocortex; HPC, hippocampus; THAL, thalamus. N = 3 mice/group. One-way ANOVA: F (3, 8) = 229.9, P < 0.0001 [Aβ (6E10) immunohistochemistry]; F (3, 8) = 94.10, P < 0.0001 (cyano-PiB). Brackets indicate differences of P < 0.05

Brain sections were processed for total Aβ load using Aβ immunohistochemistry as described above and imaged using light microscopy (see Fig. 1). A set of adjacent sections was processed for mature fibrillar amyloid deposits (avoiding the formic acid pre-treatment) using the fluorescent amyloid binding dye cyano-Pittsburgh compound-B (cyano-PiB) [56] and imaged using epifluorescence microscopy. Digital images of stained sections were acquired at 4× magnification to create a composite of the cerebral cortex and hippocampus for each section. Percent area coverage of labeled deposits was then calculated by thresholding positive signal and determining the percent of cerebral cortical tissue occupied by plaques using modified algorithms in the FIJI Open-Source image analysis software adapted from ImageJ [114].

### Capillary density estimation

We defined cortical capillaries as microvessels measuring 10 µm in diameter or less. Our technical approach to measuring total number of capillaries [***Ntot(cap)***] is adapted from published reports [80, 94, 96]. The number of intersecting points (nodes) in each capillary network created a “node-segment network” where a single capillary was defined as the vessel segment between two nodes. Each node has a valence (***n***) defined by the number of vessel segments that intersect at a node. In each individual optical dissector, we first calculated the number of capillaries (**N**_**cap**_) as a function of the number of nodes (**N**_**node**_) containing each valence (***n*** with range 3–4), as described in Eq. 1:

1 $${\varvec{N}}_{{{\varvec{cap}}}} = \left( {\frac{{{\varvec{n}} - 2}}{2} \cdot {\varvec{N}}_{{{\varvec{node}}}} } \right) + 1.$$

To determine the ***Ntot(cap)*** in each optical dissector, we calculated the sum of all ***N***_***cap***_ of each valence using correction factors as previously described [80, 94, 96], and detailed below. In the present study, we counted capillaries in two cerebral hemi-sections selected from a series through the rostral-caudal extent of the mouse brain. These selected hemi-sections represented a known fraction of total sections which we determined to be 1/53.5 (***ssf***) based on tissue thickness and the rostral-caudal extent of the mouse brain. Our randomly generated optical dissector consisted of a 143.95 µm × 111.52 µm counting frame with an area (***A***_***frame***_) equal to 16,052.65 µm^2^. The average distance between positions of the optical dissector (***A***_***step***_) varied per mouse, due to differences in brain volume, and ranged from 732,656 to 1,911,707 µm in both x and y dimensions. Thus, the counting frame also represented a known fraction of the total brain hemi-section area (***asf***), defined as (***A***_***frame***_/***A***_***step***_). Finally, we examined a known fraction of the total tissue thickness using the confocal microscope (***tsf***) defined as the average height of imaging depth per mouse (***h***) divided by the total tissue thickness (***t***) accounting for shrinkage with processing, where ***h*** varied per mouse (range: 9.61–23.70 µm) and ***t*** equals 25 µm. Adjusting for these variations in fraction of sections, area, and thickness, ***Ntot(cap)*** is presented in Eq. 2:

2 $${\varvec{N}}_{{{\varvec{tot}}}} ({\varvec{cap}}) = \sum {{\varvec{N}}_{{{\varvec{cap}}}} } \cdot \frac{1}{{{\varvec{ssf}}}} \cdot \frac{1}{{{\varvec{asf}}}} \cdot \frac{1}{{{\varvec{tsf}}}}.$$

### Pericyte estimation

A similar quantification method was used to estimate the total number of pericytes using the optical dissector probe, adapted from a previous study [94], in two cerebral hemi-sections. To identify pericytes situated amongst the capillary basement membrane, we performed dual-labeling using fluorophore-conjugated LEL and the pericyte-specific PDGFRβ antibody visualized using a fluorophore-conjugated secondary antibody. As seen in Fig. 3, the clear bumps along the LEL-labeled basement membrane, coined “ghost-like cell bodies” [94], co-localize with PDGFRβ and DAPI. Using our optical dissector, we counted these “ghost-bodies” to estimate the total number of pericytes (see Fig. 3). Using the same parameters as the capillary analysis, total numbers of pericytes (**N**_**tot peri**_) were calculated using the sum of pericytes in each optical dissector (**Q**) as listed in Eq. 3:

3 $${\mathbf{N}}_{{{\mathbf{tot}} \, {\mathbf{peri}}}} = {\mathbf{SQ}}*1/{\mathbf{ssf}}*1/{\mathbf{asf}}*1/{\mathbf{tsf}}.$$

### Astrocytic end-feet contacts estimation

To estimate the contact surface of astrocytic end-feet interfacing with the endothelial basement membrane, we identified percentage overlap of AQP4-immunoreactive end-feet and STL immunosignal as measured with 2D voxels corresponding to fluorescence intensities in FIJI. We first masked the surrounding tissue around the capillary vessel to create several “iso-surfaces” of capillary per optical dissector [40]. This allowed for detection of AQP4-labeled interaction at the level of the capillary iso-surface which could be quantified as both red- and green-fluorescent signal. Then, we took the percentage of AQP4-immunoreactive astrocytic end-feet as a function of STL (vessel) co-localizing within the given unmasked iso-surface, and created an average surface area overlap using a 2D reconstruction, as described in Eq. 4:

4 $$\text{\%}\;{\text{contact}}\;{\text{surface}} = {{{\text{AQP}}4\;{\text{stain}}\;{\text{intensity}}} \mathord{\left/ {\vphantom {{{\text{AQP}}4\;{\text{stain}}\;{\text{intensity}}} {{\text{STL}}\;{\text{stain}}\;{\text{intensity}}}}} \right. \kern-\nulldelimiterspace} {{\text{STL}}\;{\text{stain}}\;{\text{intensity}}}}.$$

### Statistical analysis

We report all data as mean ± SEM. Excel (Microsoft) and Prism software (GraphPad, San Diego, CA) were used in generating statistics and graphs for all data. The mean value per randomly sampled optical dissector was used in all statistical analyses. One-way ANOVA with Tukey’s post hoc testing was used to assess the plaque load data. Two-way ANOVA with Sidak’s multiple test comparison were used to analyze the astrocyte, capillary, and pericyte data. Using an alpha of 0.05, results were considered significant when p < 0.05.

## Results

### Cortical Aβ plaque load in Tg mice after bTBI or Sham surgery

Tg-bTBI and Tg-Sham groups had statistically significantly higher Aβ pathology loads at 12-months survival (i.e., 15-month-old mice) compared to 3-months survival (i.e., 6-month-old mice) when assessed by Aβ immunohistochemistry (Fig. 1A–F) or cyano-PiB histofluorescence stain of fibrillar Aβ deposits (Fig. 1G). No statistically significant differences in Aβ-immunoreactive plaque load in the cerebral cortex were detected between the two Tg groups at 3-months survival (Tg-bTBI/3-months: 0.2706 ± 0.0757; Tg-Sham/3-months: 0.1688 ± 0.0850) or at 12-months survival (Tg-bTBI/12-months: 5.8820 ± 0.3933; Tg-Sham/12-months: 5.4390 ± 0.5910) (Fig. 1F). Similarly, no statistically significant differences in cyano-PiB positive plaque load in the cerebral cortex were detected between the Tg-bTBI and Tg-Sham groups at 3-months survival (Tg-bTBI/3-months: 0.2087 ± 0.0326; Tg-Sham/3-months: 0.1352 ± 0.0501) or at 12-months survival (Tg-bTBI/12-months: 5.0539 ± 0.6457; Tg-Sham/12-months: 4.4579 ± 0.6931) (Fig. 1G).

Aβ-immunoreactive and cyano-PiB plaque loads in the hippocampus were higher at 12-months survival compared to 3-months survival (Aβ: Tg-Sham/3-months: 0.4797 ± 0.1040; Tg-Sham/12-months: 5.0546 ± 1.2075; Tg-bTBI/3-months: 0.4285 ± 0.1665; Tg-bTBI/12-months: 4.8861 ± 0.7188; cyano-PiB: Tg-Sham/3-months: 0.4279 ± 0.2631; Tg-Sham/12 months: 4.7037 ± 0.9840; Tg-bTBI/3-months: 0.5441 ± 0.3897; Tg-bTBI/12 months: 4.5529 ± 0.8166). No statistically significant differences in hippocampal Aβ-immunoreactive or cyano-PiB plaque loads were detected between the Tg-bTBI and Tg-Sham groups at either survival interval.

No Aβ immunoreactive or cyano-PiB fluorescent plaques were detected in brain tissue sections from Wt mice exposed to bTBI or to the Sham procedure.

### Astrocyte AQP4-immunoreactive end-foot contacts with microvasculature in Wt and Tg mice after bTBI or Sham exposure

**Fig. 2:**
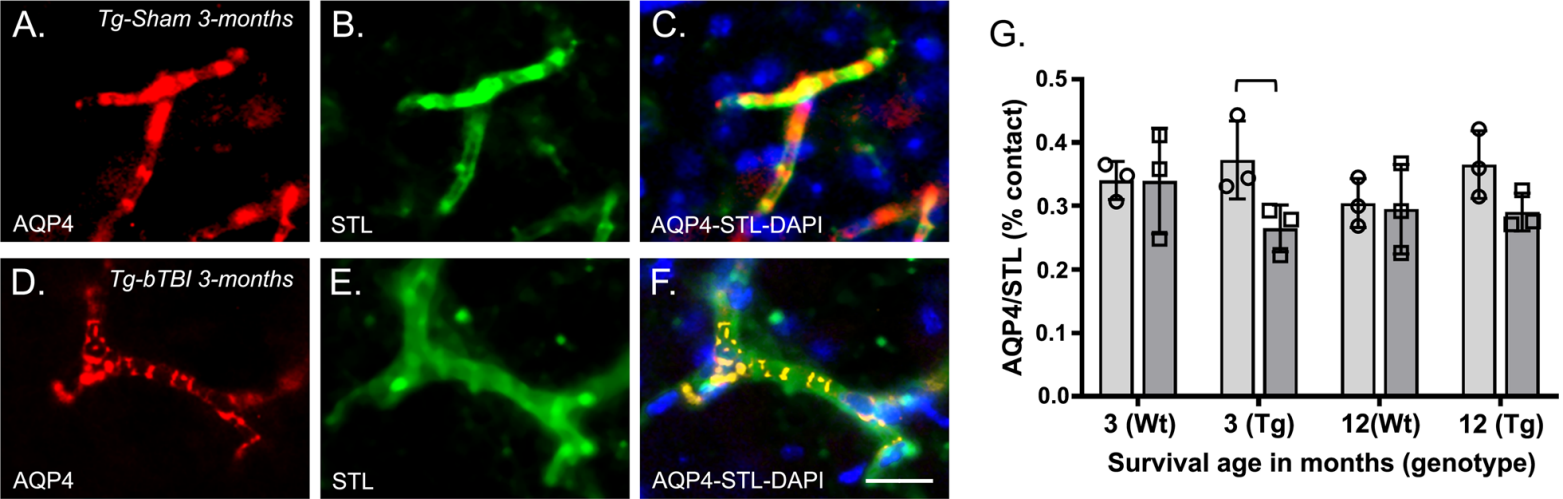
Reduced perivascular AQP4-expressing astrocytic end-feet after bTBI in APP/PS1 mice. **A**, **B** Maximum intensity projection of a confocal image stack demonstrating aquaporin-4 immunofluorescence signal (AQP4, red) contacting capillaries labeled with a fluorescent lectin (STL, green) in representative Tg-Sham (**A**–**C**) and Tg-bTBI (**D**–**F**) mice at 3-months survival (n = 3 mice/group). Merged images (**C**, **F**) have an overlay of DAPI-labeled nuclei (blue). **G** Quantification of percent area coverage of sampled AQP4 immunofluorescence interfacing with the STL-labeled endothelium. N = 3 mice/group. Two-way ANOVA: F (1, 16) = 5.816, P = 0.0343. Brackets indicate differences of P < 0.05. Scale bar = 20 µm (**A**–**F**)

AQP4 immunofluorescence was observed primarily in contact with capillary endothelium, indicating its preferential localization to astrocytic end-feet (Fig. 2A–F). Quantification of the interface between AQP4-immunofluorescence at the level of capillary endothelium showed statistically significantly lower values in the Tg-bTBI group compared to the Tg-Sham group at 3-months survival (Fig. 2G) but not at 12-months survival. We did not observe differences in AQP4 among experimental groups of Wt mice at either survival interval (Fig. 2G).

### Numbers of pericytes in Wt and Tg mice after bTBI or Sham exposure

**Fig. 3:**
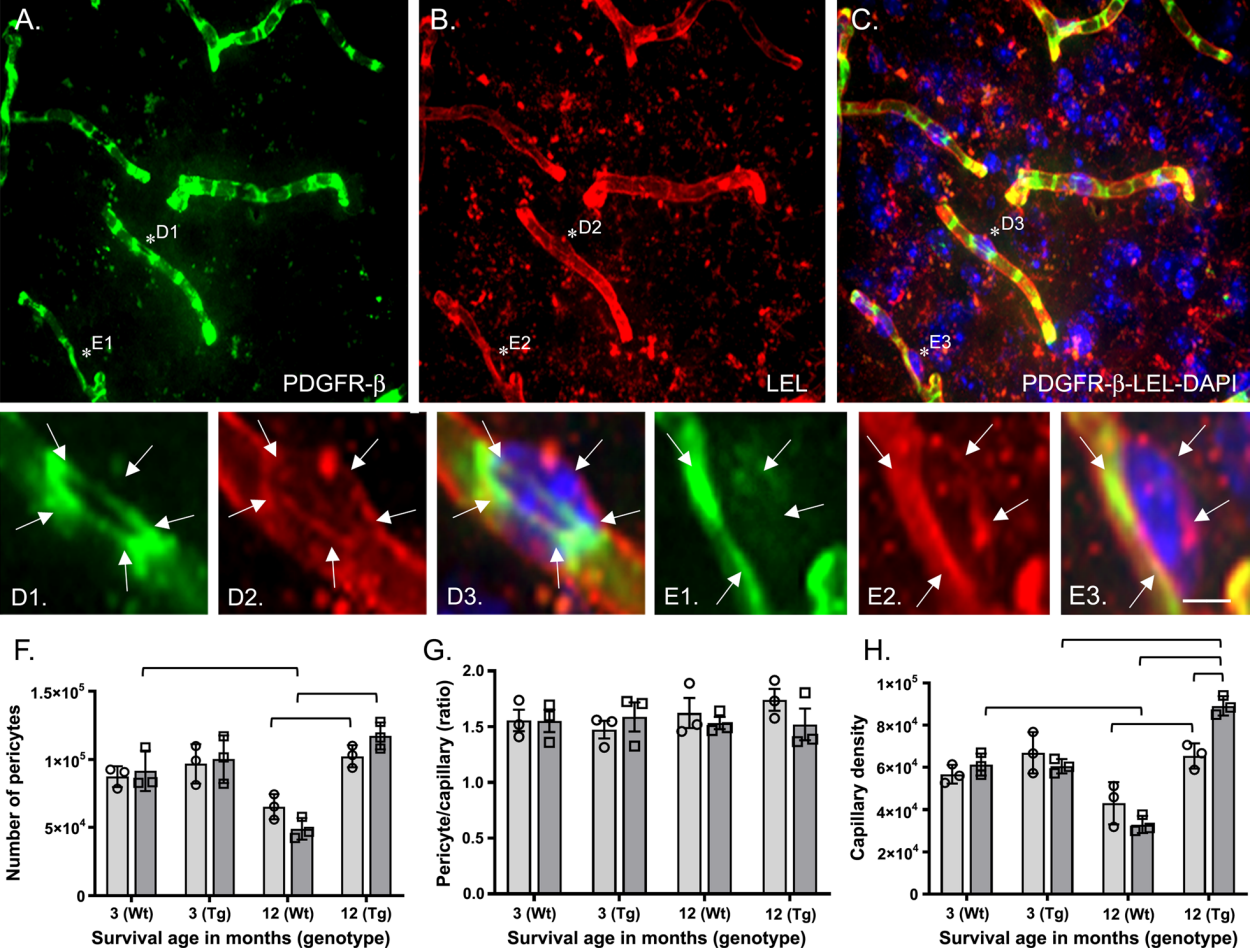
Blast TBI affects pericytes and capillaries differently in APP/PS1 mice and wild type mice. **A**–**E** Maximum intensity projection of a confocal image stack demonstrating PDGFR-β immunofluorescent signal (**A**, **D1**, **E1**, green) associated with capillaries labeled with a fluorescent lectin (**B**, **D2**, **E2**, LEL, red). Merged pairs of images with DAPI-stained nuclei are shown in panels **C**, **D3**, and **E3**. Asterisks in panels **A**–**C** mark the position of two pericytes shown at higher magnification in panels **D1**–**D3** and **E1**–**E3** where arrows demarcate the area occupied by each pericyte. **F**–**H** Graphs showing total numbers of pericytes (**F**), pericytes per capillary length (**G**), and capillary densities (**H**) in experimental groups. N = 3 mice/group. Two-way ANOVA (pericytes): F (3, 16) = 22.15, p < 0.0001 (row). Two-way ANOVA (capillaries): F (3, 16) = 38.98, P < 0.0001 (row); F (1, 16) = 3.299; P = 0.0297 (column); F (3, 16) = 8.448, P = 0.0014 (interaction). Brackets indicate differences of P < 0.05. Scale bar = 25 µm (**A**-**C**)﻿ 5 µm (**D1**-**D3**, **E1**-**E3**)

Figure 3 illustrates representative images of immunofluorescently labeled pericytes (anti-PDGFRβ, green fluorescence, panels A, D1, E1, D3, E3) located within bump-like protrusions of the basement membrane (LEL, red fluorescence, B, D2, E2, D3 E3). There were no injury- or genotype-related effects on pericyte numbers at 3-months survival (Fig. 3F).

The Wt-bTBI group at 12-months survival had statistically significantly lower numbers of pericytes compared to the Wt-bTBI group at 3-months survival (Fig. 3F). In Tg mice, pericyte numbers were not different between the Tg-bTBI group and the Tg-Sham group at 3-months or 12-months survival. There were no differences when the Tg-TBI and Tg-Sham groups at 3-months survival were compared to the same groups at 12-months survival (Fig. 3F). However, significantly greater numbers of pericytes were observed in Tg mice compared to Wt mice for both the bTBI and Sham groups at 12-months survival (Fig. 3F).

### Pericytes relative to capillary density in Wt and Tg mice after bTBI or Sham exposure

Figure 3G shows the total number of pericytes relative to capillary density within each experimental group. No differences in pericyte numbers were observed between experimental groups when adjusted for capillary densities (Fig. 3G).

### Capillary densities in Wt and Tg mice after bTBI or Sham exposure

In Wt mice, the bTBI and Sham groups did not differ by capillary density at 3-months survival or at 12-months survival (Fig. 3H). The Wt-bTBI group at the 12-months survival had statistically significantly lower capillary density when compared to the Wt-bTBI group at 3-months survival (Fig. 3H).

In Tg mice, capillary density at 12-months survival was statistically significantly higher in the bTBI group than the Sham group (Fig. 3H). The Tg-bTBI group had significantly higher capillary density at 12-months survival than at 3-months survival (Fig. 3H).

Numbers of capillaries did not differ between genotypes at 3-months survival (Fig. 3H). At 12-months survival, both the Tg-bTBI group and the Tg-Sham group had statistically significantly higher numbers of capillaries compared to the Wt-bTBI group and the Wt-Sham group, respectively (Fig. 3H).

## Discussion

This study demonstrates chronic changes in the cerebral cortical microvasculature after a single bTBI (~ 138 kPa/~ 20 psi) in adult female transgenic (Tg) APP/PS1 human Aβ over-producing mice compared to non-transgenic wild type (Wt) mice which have physiological levels of murine Aβ. We observed that compared to the Sham procedure, bTBI resulted in lower AQP4-expression at contact areas between astrocyte end-feet and the capillary network in the cerebral cortex of Tg, but not Wt, mice at 3-months survival. At 12-months survival, Tg mice exposed to bTBI had greater capillary densities in the cerebral cortex than Tg mice exposed to the Sham procedure (12-months survival) as well as Tg mice exposed to bTBI and assessed at 3-months survival. In contrast, Wt mice exposed to bTBI had statistically significant lower capillary densities at 12-months survival than at 3-months survival. Changes in microvascular densities paralleled changes in numbers of pericytes in both Tg and Wt mice, resulting in stable numbers of pericytes per capillary density at both chronic survival intervals after bTBI or Sham procedure.

Our previous study found that APP/PS1 Tg mice subjected to a bTBI at 2–3 months of age and assessed after a 2-months survival had no significant changes in cortical Aβ plaque load relative to their Sham-exposed Tg counterparts [44]. Similarly, in the current study using the same bTBI injury procedure, we did not detect statistically significant changes in Aβ load after a 3-months survival in APP/PS1 Tg-bTBI mice compared to Tg-Sham mice. These results are in agreement with a recent report that Aβ plaque load is not significantly different in 5-months-old APP/PS1 Tg mice exposed to repetitive low-level bTBI compared to sham APP/PS1 Tg mice when assessed after 3–4 months survival [100]. We extend these findings by showing that at a longer chronic (12-months) survival interval, bTBI has no significant effect on Aβ load in APP/PS1 Tg mice. Interestingly, a study of bTBI in wild type rodents with acute (24 h and 1 week) survival reported no changes in endogenous Aβ concentration when the level of blast exposure was similar to our study (116.7 kPa), while reduced Aβ42 concentrations were detected when using lower level (36.6 kPa and 74.5 kPa) blast exposures [27]. The same group reported that in APP/PS1 Tg mice, low-level (34.5 kPa) blast exposure had no effect on soluble and insoluble Aβ42 at 1 week survival while Aβ42 levels were reduced at 1-month survival [100]. Collectively, these studies indicate that the level/severity of bTBI, in addition to other variables such as the number and frequency of blast exposures and the length of survival, may differentially influence Aβ concentrations in brain. However, the progressive transgene-driven Aβ deposition in Tg mice could mask the potentially subtle injury-related changes in cortical Aβ deposits. It will be important that future studies examine time points between 3 and 12 months.

While bTBI significantly altered AQP4 expression at the astrocytic end-foot/endothelium interface in Tg mice, we did not observe such changes in the age-matched Wt mice undergoing the same injury protocol. This observation suggests that high concentrations of human Aβ present in the Tg mouse brain confers susceptibility for chronic dysfunction of perivascular astrocytes in the context of bTBI exposure. Astrocyte dysfunction has been reported in multiple models of CNS insult. For example, after TBI, ischemia, and in neurodegenerative disease, astrocytes enter a hypertrophic state characteristic of astrogliosis [31, 117], and this process can contribute to impairments in gliovascular connections, BBB permeability, and cerebral blood circulation [21, 97, 98]. Several studies reported adverse effects of bTBI on perivascular astrocytes in adult male rats. Acutely after single or repetitive bTBI using an open-ended shock tube model (peak pressure of 150 kPa), Uzunalli and colleagues reported reactive astrogliosis accompanied by early signs of astrocytic end-foot displacement from blood vessels, and a trend for reduced AQP4 expression [127]. Another bTBI study applied a series of three low-energy blast exposures of 74.5 kPa (10.8 psi) intensity and reported reduced levels of vascular-associated astrocytic GFAP, coupled with swelling of perivascular astrocytes and degeneration of their end-feet, 6-weeks after the last impact [36]. Our current data suggest that in wild type animals which do not accumulate human Aβ, astrocyte pathology, reported previously at acute and subacute time points after TBI, may resolve over chronic time intervals after injury. Alternatively, sex or species differences may account for discrepancies between studies. When bTBI occurs in individuals with ongoing Aβ accumulation as a predisposition for developing AD-like amyloid pathology (modeled in adult Tg APP/PS1 mice exposed to bTBI), perivascular astrocyte dysfunction is either more severe or damaged astrocytes undergo slower recovery relative to astrocytes in Wt mice. The consequences of chronic astrocyte changes (pathological changes and recovery) on microvascular circulation and BBB permeability after bTBI require further exploration.

The current study finding that the longer bTBI survival in Wt mice (12-months compared to 3-months) is associated with lower numbers of PDGFR-β immunolabeled pericytes could reflect a progressive loss of pericytes lengthily after bTBI. Loss of PDGFR-β expression was reported over 24 h after a bTBI in rats [2]. However, single or repetitive exposure of rats to bTBI did not affect numbers of PDGFR-β-immunoreactive pericytes at 1–3 days survival [127]. Using CD-13 and desmin immunofluorescence as alternative markers of pericytes, the same study confirmed stability of pericyte numbers after a single bTBI exposure but detected a transient increase in pericyte numbers after repetitive bTBI [127]. These data indicate that different models of bTBI and use of different markers of pericytes can result in different and even opposite observations when pericyte numbers are assessed acutely after bTBI in rodents. In other models of TBI, loss of pericytes, a disrupted (permeable) BBB, and gliosis within days after TBI (i.e., acutely) were reported in wild type C57Bl/6 mice exposed to closed head mild controlled cortical impact (CCI) [133] and in rats with fluid percussion injury to the exposed brain dura [12]. Another study, using the model of CCI injury over the exposed brain dura of wild type C57Bl/6 mice, reported loss of pericytes within 6–12 h after injury followed by a recovery and proliferation of pericytes by 5 days after injury [135]. Loss or dysfunction of pericytes can affect microvascular flow [15] and BBB permeability [11]. It has been hypothesized that pericyte loss “would disrupt optimized flow patterns and cause imbalanced perfusion and oxygen delivery” [45]. This imbalance could be exacerbated by injury- or disease-induced elevations in reactive oxygen species, which cause pericyte contraction [45], or by exposure to high levels of human Aβ forms [45] which has been associated with pericyte contraction in AD and mouse models of the disease [8, 70, 95]. In regard to BBB permeability, pericytes influence the transcription of tight junction proteins by endothelial cells [11, 26]. Changes in BBB permeability coinciding with loss of expression of the tight junction protein claudin-5 in the hippocampus of adult male C57Bl/6J mice were reported up to 72 h after a 20-psi bTBI [78], and loss of occludin acutely was reported in rat models of single bTBI [63] and repeated bTBI [48]. Another bTBI study in rat [2] reported diminished expression of tight junction proteins in parallel with loss of PDGFR-β-immunoreactivity. Though analysis of tight junction proteins was outside the scope of the current study, whether such changes persist lengthily and are influenced by changes in pericytes are important avenues of investigation.

In addition to impairing the NVU, loss or dysfunction of pericytes after bTBI exposure could also influence Aβ pathology. For example, when the Tg APPsw mouse model of human Aβ peptide overproduction was crossed with pericyte-deficient PDGFR-β+/− mice, accelerated Aβ accumulation in brain parenchyma and in blood vessel walls was observed [111]. We did not see statistically significant effects of bTBI on pericyte numbers in our Tg APP/PS1 mice compared to Tg-Sham mice. This was surprising when considering previous reports of pericyte depletion, dysfunction, and/or disrupted pericyte-endothelial cell signaling with exposure to soluble toxic Aβ peptides due to their impaired clearance and accumulation [15, 19, 24, 28, 111]. In relation to Aβ pathology in postmortem AD brains, several studies reported reduced numbers of pericytes and pericyte coverage of capillaries which corresponded to greater Aβ deposition and BBB impairment in these cases [42, 87, 115], while others reported no significant changes in pericyte numbers [32, 33, 119]. The lack of a difference in the number of pericytes per capillary density when comparing bTBI and Sham groups of our Tg mice at 12-months survival was likely due to higher pericyte frequencies and capillary densities, as both could be stimulated by Aβ. Proliferation of pericytes contributes to increased angiogenesis [106] and, alternatively, angiogenesis could influence recruitment of pericytes to newly formed capillaries [18]. It will be important to determine if these cells function normally, particularly in a brain environment with high concentrations of Aβ which causes capillary constriction at pericyte locations [95].

Our observations of higher capillary densities at 12-months survival than 3-months survival in Tg mice exposed to bTBI, and no net change in pericyte numbers per capillary density are reminiscent of the results from stereological analyses in the frontal cortex of AD patients, reporting a 24% increase in capillary density and no change in numbers of pericytes per capillary segment [33]. Other studies also reported that Aβ pathology is associated with higher microvascular density in AD brain [14, 22, 33, 85, 101, 107] and in aged transgenic mice with human tau-overexpression [17]. This could be due, in part, to angiogenesis which plays a role in compensatory vascular remodeling after cerebral hypoperfusion and hypoxia and/or an inflammatory process activated by AD pathology [102, 128]. Angiogenic signaling factors are increased in brains of people with AD and vascular dementia [17, 102, 125], and multiple serum biomarkers of vascular functions including angiogenesis (vascular endothelial growth factor; von Willebrand factor) are elevated after experimental TBI [7, 73, 112]. Thus, angiogenesis may underlie the increased microvascular densities observed in Tg mice in our bTBI study and reported previously in AD, but this change may not be successful in countering long-term brain hypoxia because, similar to the aftermath of stroke and in AD, newly formed microvascular networks can have impaired vessel reactivity and reduced lumen diameter which manifests as greater vasoconstriction, thus limiting oxygen delivery [22, 125]. From a therapeutic standpoint, stimulation of angiogenesis by brain implantation of human umbilical cord perivascular cells after fluid percussion TBI in rats normalized (the reduced) capillary densities and pericyte coverage of capillaries, resulting in lower BBB permeability and less axonal injury [12]. In contrast to the findings in Tg mice, we observed that Wt mice with bTBI had lower capillary density at 12-months survival than after 3-months survival, possibly reflecting an effect of age on capillary density in Wt mice as has been reported previously [113], or lack of high Aβ concentrations which could stimulate angiogenesis and pericyte proliferation in Tg mice.

Some limitations of the current study should be considered. The number of mice per experimental group was small, however we used an unbiased stereological approach with five optical dissectors per brain hemi-section and, overall, 10 sites per mouse and did not observe large variations within groups. Nevertheless, we recognize that the study was not sufficiently powered to draw definitive conclusions. The high complexity of the NVU requires that future studies of bTBI in mouse models of human neurogenerative diseases analyze additional components of the microvasculature and BBB including the endothelial tight junction proteins. TBI can impair the ability of astrocytes and pericytes to crosstalk with endothelial cells and this can lead to loss of tight junction proteins and further impairment of the BBB permeability and capillary flow [10, 11, 54, 121]. Studies of human Aβ-overexpressing transgenic mice with longer survival intervals after bTBI will further define chronic changes in the microvasculature, permeability of the BBB, and cerebral blood circulation in relation to progressive accumulation of Aβ as a risk of developing chronic neurodegeneration.

## Conclusions

Human and experimental studies of brain injury report changes in BBB permeability, induction of perivascular inflammation, altered endothelial-immune cell interactions, and dysfunction of endothelial cells and pericytes [4]. While most of these changes are reported within the acute and subacute period, the current study contributes to a better understanding of the chronic phase of bTBI in relation to a risk of developing sustained cerebral vascular dysfunction and AD-related Aβ pathology. We demonstrate that at the level of the microvasculature, bTBI results in significant long-term changes (i.e. loss of pericytes and lower capillary density) in Wt mice while more complex detrimental and potentially compensatory chronic changes occur in a transgenic mouse model with AD-relevant brain accumulation of human Aβ. In the latter model, we observed that bTBI resulted in chronic loss of astrocytic AQP4 expression at the level of microvessels which could be due to AQP4 redistribution from end-feet to the soma, retraction of astrocytic end-feet, or lack of coverage of newly formed vessels. Our observation that chronic bTBI in transgenic AD mice is associated with increased capillary density, with no significant change in numbers of pericytes relative to capillary density, is similar to what has been reported in brains of AD patients [33]. Further efforts to clarify the mechanisms responsible for chronic changes in components of the microvasculature after bTBI in relation to increased Aβ accumulation may help identify areas for early or delayed therapeutic intervention.

## Data Availability

The datasets used and/or analyzed during the current study are available from the corresponding author on reasonable request.

## Abbreviations

Aβ: Amyloid beta
AD: Alzheimer’s disease
APP: Amyloid precursor protein
AQP4: Aquaporin-4
BBB: Blood–brain barrier
bTBI: Blast-induced traumatic brain injury
CTE: Chronic traumatic encephalopathy
IP: Intraperitoneal
LEL: Lycopersicon Esculentum
NVU: Neurovascular unit
PDGF: Platelet derived growth factor
PDGFRβ: Platelet derived growth factor receptor beta
PiB: Pittsburgh compound B
PS1: Presenilin 1
STL: Solanum Tuberosum
Tg: Transgenic
TBI: Traumatic brain injury
Wt: Wild type
